# Comparative Metagenomics Reveals Microbial Diversity and Biogeochemical Drivers in Deep-Sea Sediments of the Marcus-Wake and Magellan Seamounts

**DOI:** 10.3390/microorganisms13071467

**Published:** 2025-06-24

**Authors:** Chengcheng Li, Bailin Cong, Wenquan Zhang, Tong Lu, Ning Guo, Linlin Zhao, Zhaohui Zhang, Shenghao Liu

**Affiliations:** 1 Marine Ecology Research Center, First Institute of Oceanography, Ministry of Natural Resources, Qingdao 266061, China; lichengcheng@fio.org.cn (C.L.); biolin@fio.org.cn (B.C.); lutong@fio.org.cn (T.L.); zhaolinlin@fio.org.cn (L.Z.); zhangzhaohui@fio.org.cn (Z.Z.); 2 School of Advanced Manufacturing, Fuzhou University, Jinjiang 362200, China; guoning@fio.org.cn; 3 National Deep Sea Center, Ministry of Natural Resources, Qingdao 266237, China; zhangwq@ndsc.org.cn

**Keywords:** Proteobacteria, metagenome-assembled genomes, sulfur oxidation, metal resistance genes, microbial interactions

## Abstract

Seamounts are distributed globally across the oceans and are generally considered oases of biomass abundance as well as hotspots of species richness. Diverse microbial communities are essential for biogeochemical cycling, yet their functional partitioning among seamounts with geographic features remains poorly investigated. Through metagenomic sequencing and genome-resolved analysis, we revealed that Proteobacteria (33.18–40.35%) dominated the bacterial communities, while Thaumarchaeota (5.98–10.86%) were the predominant archaea. Metagenome-assembled genomes uncovered 117 medium-quality genomes, 81.91% of which lacked species-level annotation, highlighting uncultured diversity. In the Nazuna seamount, which is located in the Marcus-Wake seamount region, microbiomes exhibited heightened autotrophic potential via the 3-hydroxypropionate cycle and dissimilatory nitrate reduction, whereas in the Magellan seamounts regions, nitrification and organic nitrogen metabolism were prioritized. Sulfur oxidation genes dominated Nazuna seamount microbes, with 33 MAGs coupling denitrification to sulfur redox pathways. Metal resistance genes for tellurium, mercury, and copper were prevalent, alongside habitat-specific iron transport systems. Cross-feeding interactions mediated by manganese, reduced ferredoxin, and sulfur–metal integration suggested adaptive detoxification strategies. This study elucidates how deep-sea microbes partition metabolic roles and evolve metal resilience mechanisms across geographical niches. It also supports the view that microbial community structure and metabolic function across seamount regions are likely influenced by the geomorphological features of the seamounts.

## 1. Introduction

The deep sea encompasses various geographic structures such as hydrothermal vents, cold seeps, seamounts, trenches, abyssal plains, and mid-ocean ridges [1]. These ecosystems exhibit distinctive traits under extreme conditions such as lack of light, low temperatures, high pressure, and nutrient shortages, making them attractive settings for biological and geological research [2,3,4]. Deep-sea seamounts have heterogeneous environmental factors, such as geographic isolation, limited spatial range, and fast water flow, all of which contribute to the development of varied biological communities. Seamount ecosystems are hotspots of biodiversity and significantly influence marine species distribution and ecological dynamics. Seamount slopes frequently feature volcanic rocks, which are commonly adorned with layers of ferromanganese crusts [5]. The western Pacific Ocean harbors several seamounts rich in polymetallic nodules and crusts, such as the Magellan seamount region [6,7]. Polymetallic nodules contain valuable metals like nickel, copper, cobalt, and manganese, essential for modern industrial needs, and are under consideration for deep-sea mining to support the shift towards sustainable energy sources [8,9].

Microorganisms, as primary producers, are central to chemoautotrophic processes, which involve the creation, alteration, and decomposition of marine organic matter. These primary producers are vital for the continuous supply of materials and energy to the marine environment [10,11]. The deep-sea sediments are teeming with a diverse array of microorganisms that are essential for sustaining biogeochemical cycles [12]. The deep-sea halophilic bacterium, *Halomonas* sp. MNB13, has been isolated from these nodules. This bacterium can withstand high levels of manganese ions and is involved in the regulation of manganese mineralization through cysteine metabolism, with the production of H_2_S influencing the oxidation level of manganese [13]. Furthermore, *Georhizobium* sp. MAB10, derived from manganese nodules, can link manganese oxidation to anaerobic photosynthesis, facilitating its growth. This strain features an anaerobic photosynthetic system that captures near-infrared light to produce ATP and concurrently forms insoluble manganese oxides [14]. However, due to sampling difficulties and the challenges of culturing deep-sea microbes in laboratories, the microbial diversity, community interaction along environmental gradients and microbially driven biogeochemical cycling in deep sea are still far from fully understood [11,15].

Currently, metagenomic sequencing is frequently employed to explore the genetic diversity and prevalence linked to nitrogen, sulfur, carbon, and phosphorus metabolism among microbial communities [16,17]. Understanding the diversity of microorganisms and their interaction is essential for uncovering the vertical stratification and succession of intricate microbial communities within marine sediments [18]. The structure of microbial communities and their metabolic potential in sediments of seamounts in the western Pacific remain insufficiently explored. Further research is imperative to establish a foundational understanding of these communities and their responses to environmental conditions, which is crucial for the conservation of marine ecosystems, particularly those abundant in polymetallic nodules [19]. In addition, deep-sea microbial genetic resources represent new frontiers for bioprospecting, as these microbes have evolved unique molecular adaptations to survive extreme conditions, resulting in the production of distinctive enzymes and secondary metabolites. These biomolecules, which support essential physiological and ecological functions, also offer significant potential for developing novel products across industries such as pharmaceuticals, cosmetics, and food supplements [20]. The unculturability of deep-sea microbes, prevalent across diverse deep-sea ecosystems, challenges our capacity to characterize microbial diversity and decipher metabolic interactions using conventional culturomics approaches. Metabolic interactions are the threads holding a community of microbes together [11,21,22].

Whether seamounts function as isolated ecosystems fostering endemic faunas and their connectivity issues have long been research priorities [23,24]. Many scientists support the view that seamounts have a certain degree of connectivity [23]. However, the perspective has been disputed by studies of animal taxa with limited migratory capacity, showing low connectivity between seamounts with high endemism at a local level [25]. Therefore, it is imperative to acquire data on microbial communities in diverse seamount ecosystems and compare their biogeochemical cycling capacities, which may also offer novel insights into this controversial topic.

In this study, we collected two surface sediments from the Nazuna seamount in the Marcus-Wake seamount region, and five surface sediments from the Magellan seamount region. Through genome-resolved metagenomics, this study specifically aims to (1) define the core microbiome and investigate the potential microbial diversity, (2) investigate microbiome structure, function, and activity, and (3) identify links between seamount microbiomes. We compared distinct differences in microbial community composition between two seamount regions, revealing functional gene distribution related to sulfur, nitrogen, and metal metabolism, and their link to environmental factors. Metabolic network analysis highlighted key interactions between sulfur-metabolizing bacteria and other microorganisms, emphasizing the crucial role of metal elements. These findings uncover habitat-driven metabolic specialization and adaptive strategies (e.g., nitrogen–sulfur coupling, metal resistance) in deep-sea microbes, advancing our understanding of their roles in biogeochemical cycling sustaining deep-sea ecosystem functions.

## 2. Materials and Methods

### 2.1. Site Description and Sample Collection

**Figure 1:**
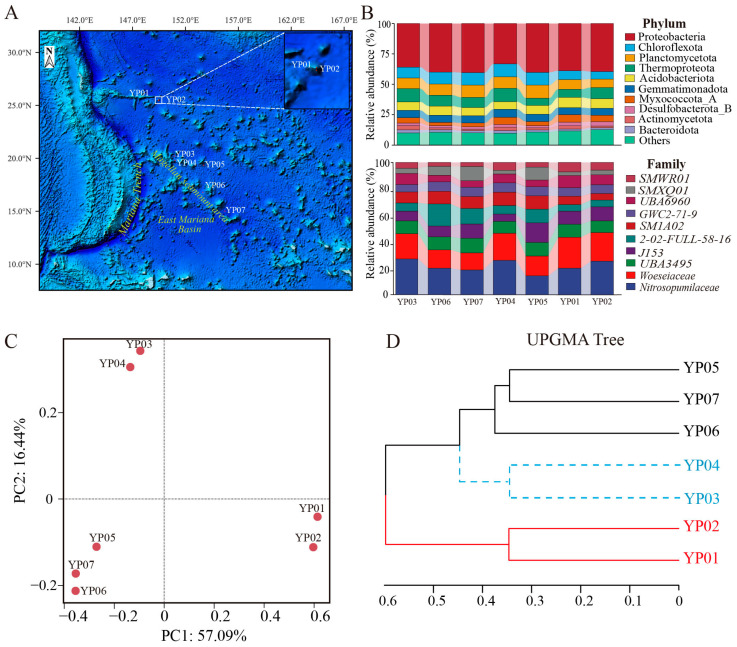
Distribution of sampling sites and comparisons of microbial composition among seven samples. (**A**) Sampling sites in the study region from the western Pacific Ocean, marked by red circles dots. (**B**) Relative abundance of dominant microorganisms at the phylum and family level in seven samples. (**C**) Principal Coordinates Analysis (PCoA) was performed based on the taxonomic and functional annotation of seven metagenomic data to reveal the differences in microbial community composition among the samples. (**D**) Community similarities among the samples were evaluated through UPGMA clustering with metagenome reads.

Sediment samples were collected from the western Pacific Ocean during multiple survey missions aboard a research vessel (RV) between September 2023 and June 2024. The seamount named Nazuna has never been studied, and our research initiative specifically selected this seamount as its primary study subject. YP01 and YP02 sediments were collected from Nazuna seamount. Five deep-sea sediments from the Magellan seamount area (YP03, YP04, YP05, YP06, and YP07) were obtained using a box corer, and seamount sediments were collected by China’s Jiaolong submersible (Figure 1A). Each sediment was immediately transferred to sterile plastic containers and stored at −80 °C until the extraction of DNA.

### 2.2. DNA Extraction and Sequencing Analysis

Genomic DNA was extracted three times from each sample using the Qiagen DNeasy^®^ PowerSoil Pro Kit following the manufacturer’s protocol (Qiagen, Hilden, Germany) and pooled into one sample. The integrity of the extracted DNA was evaluated via agarose gel electrophoresis, and its concentration and purity were measured with a Nanodrop 2000 spectrophotometer (Thermo Fisher Scientific, Waltham, MA, USA) and a Qubit 4 Fluorometer (Thermo Scientific, USA). Illumina sequencing libraries were prepared with the NEBNext Ultra DNA Library Prep Kit (New England Biolabs, Ipswich, MA, USA). Sequencing was conducted on the Illumina NovaSeq™ X Plus platform by Beijing Novogene Technology Co., Ltd. (Beijing, China) Initial assembly of raw sequencing reads was performed using FLASH (v1.2.11) to merge paired-end reads into contiguous sequences [26]. Subsequently, stringent quality control was applied with fastp (version 0.23.1) to filter out low-quality reads and obtain high-quality clean reads [27].

### 2.3. Read-Based Phylogenetic Annotation

The taxonomy of clean reads for each sample was determined using Kraken2 with a customized database containing bacteria, archaea, fungi, viruses, protozoa, and algae genome sequences from the NCBI RefSeq database (release number: 20221209). Reads were classified into seven phylogenetic levels (domain, phylum, class, order, family, genus, species), or remained unclassified. The abundances of taxa were estimated using Bracken, which provides accurate estimates at the species and genus levels, even in cases of multiple near-identical species. The relative abundance at a certain level in this study represents the total abundance of species belonging to that level.

### 2.4. Gene Function Annotation Based on Unique-Gene

Clean reads from each sample were processed with MegaHit (v1.1.1-2-g02102e1) using the parameter “--min-contig-len 500” to produce contigs. Open reading frames (ORFs) were predicted from these contigs using METAProdigal (version 2.6.3) and clustered to generate a unique gene set through CD-HIT [28] with specific parameters (-n 9 -c 0.95 -G 0 -M 0 -d 0 -as 0.9 -r 0 -T 80). The unique gene set underwent searches against KEGG databases using kofamv1.2.0 for protein function annotations. Additionally, comparisons were made with the Carbohydrate-Active Enzymes (CAZy version 8) database, the eggNOG v5.0 database, and the NCBI NR database through DIAMOND [v0.9.22.123].

Metagenome reads were annotated using the Kyoto Encyclopedia of Genes and Genomes (KEGG) (http://www.kegg.jp, accessed on 15 January 2025) database. Gene functional annotations like COG and CAZyme were assigned utilizing eggNOG-mapper BLASTP search in eggNOG 5.0 [29] with specific criteria (e-value ≤ 1 × 10^−3^ and score ≥ 60). Salmon was applied with default parameters [30] to map clean reads back to predicted genes in each sample and determine gene abundances [31]. Moreover, functional gene annotations related to nitrogen, sulfur, iron, and enzymes were carried out using Diamond (version 0.8.35) against NR, NCycDB, SCycDB, and FeGenie databases. Enzymes involved in glycosidic bond degradation, modification, or formation were annotated using the Carbohydrate-Active Enzymes (CAZy) database. Individual metagenomic libraries’ contigs were binned with MetaWRAP based on tetranucleotide frequency and coverage values to identify potential microorganisms engaged in biogeochemical cycles.

### 2.5. Metagenome Sequence Assembly, Binning and Genome Annotations

Metagenome assembly was conducted with MEGAHIT (version 1.1.2), excluding contigs smaller than 500 bp [32]. The quality of the assembly was evaluated using QUAST [33], and redundancy was minimized with MMseqs2 by applying a threshold of 95% similarity and 90% coverage [34]. Binning was performed based on tetranucleotide frequency and coverage metrics on the contigs from each metagenomic library using MetaWRAP v1.3 [35]. The completeness and contamination levels of the deduplicated bins were assessed using CheckM v1.0.7 [36]. Metagenome-Assembled Genomes (MAGs) with >50% completeness and <10% contamination were selected and subsequently dereplicated using dRep v2.6.2 [37]. Taxonomic assignments were made utilizing GTDB-Tk, relying on the Genome Taxonomy Database [38]. A maximum-likelihood phylogenetic tree was constructed using 95 bacterial marker genes from GTDB-Tk. The abundance of each bin was computed using MetaWRAP’s Quant_bins module, which involved mapping reads to contigs and calculating average coverage across samples.

Gene predictions for the remaining bins were performed using Prodigal v2.6.3 with default parameters [39]. To annotate genes associated with biogeochemical cycling, the identified genes were aligned against the Kyoto Encyclopedia of Genes and Genomes (KEGG) database using KofamScan (e-value ≤ 0.001). Additionally, functional genes involved in nitrogen (N), sulfur (S), carbon (C), and arsenic (As) cycling were annotated using Diamond (v0.8.35) against the KEGG, NCBI-nr, eggNOG, and Pfam databases (e-value ≤ 1 × 10^−5^). Genes encoding proteins related to iron (Fe) cycling were annotated using FeGenie v1.0 [40,41]. Metal transport proteins within MAGs were detected through BLASTP searches against the Transporter Classification Database (TCDB) with an e-value cutoff of 1 × 10^−20^ [12].

### 2.6. Metabolic Interaction Analysis Based on Metagenomic Sequencing Data

Metabolic interaction was analyzed using metagenomic sequencing data based on the iNAP 2.0 analysis platform. To identify shared metabolites among metagenome-assembled genomes (MAGs) using the iNAP2.0 platform, we first prepared genome-scale metabolic models (GSMMs) by uploading a zip file containing the MAGs. These GSMMs were automatically constructed using Prokka for gene prediction and CarveMe for model generation. We then inferred pairwise metabolic interactions among these GSMMs using three approaches: PhyloMint to calculate competition and complementarity indices, SMETANA to assess cross-feeding interactions, and parsimonious Flux Balance Analysis (pFBA) to determine metabolic distances. These interactions were used to construct a metabolic interaction network, with thresholds determined using Random Matrix Theory (RMT). The network was analyzed for topological features, such as identifying hub nodes, and potential transferable metabolites were identified as intermediate nodes connecting microbial nodes. The results were presented in a bipartite network format, highlighting the metabolic complementarity among different MAGs [42].

### 2.7. Statistical Analyses

The overall differences in bacterial and archaeal community compositions were visualized through Principal Coordinate Analysis (PCoA) based on Bray–Curtis distances. The community composition of each sample (relative abundance > 0.7% at the phylum level) was combined with UPGMA tree based on Bray–Curtis dissimilarity using. The gene abundance was visualized with a heatmap.

## 3. Results

### 3.1. Microbial Community Composition from the Sediments in the Western Pacific

To characterize the microbial community composition and metabolic potential of microbes in the western Pacific, we conducted the metagenomic sequencing and analysis for the surface sediments from two seamount regions. Clean reads were generated by a data filter and quality control for the surface sediment samples of YP01, YP02, YP03, YP04, YP05, YP06, and YP07, ranging from 20.88 to 36.23 GB (Table S1). Considering the challenges posed by low-abundance or highly similar species, we initially performed taxonomic annotation on the gene sets of seven samples using the NR database to explore the microbial community composition (Figure 1B). Our results demonstrated that bacteria, rather than archaea, were the dominant microbial communities in all samples. Among the bacterial communities, the phyla Proteobacteria (classes *Alphaproteobacteria*, *Gammaproteobacteria* and *Zetaproteobacteria*), Chloroflexota, Planctomycetota, and Acidobacteriota were the dominant taxa across all samples. Moreover, Proteobacteria were the greatest bacterial taxa at phylum, accounting for 33.18–40.35% of the total microbial abundance in all samples. *Thaumarchaeota* was the most enriched archaeal, with relative abundances of 5.98–10.86%.

At the family taxonomic level, microbial communities across samples were predominantly represented by J153 and *Woeseiaceae* (Gammaproteobacteria), *UBA3495* (Chloroflexota), and *Nitrosopumilaceae* (Thaumarchaeota), which constituted the core microbiome (Figure 1B). Comparative analysis revealed distinct clustering patterns in community structure: YP03 and YP04 shared comparable taxonomic composition and species abundance profiles, while YP06, YP07, and YP05 formed another cluster with similar microbial distribution characteristics. Notably, the Nazuna seamount samples (YP01 and YP02) demonstrated remarkable consistency in both phylogenetic composition and quantitative microbial distribution. Beta diversity analysis employing Principal Coordinates Analysis (PCoA, 73.53% variance explained by two principal coordinate axes) and UPGMA clustering with bootstrap support values revealed significant segregation among the seven microbial communities. The ordination plot and phylogenetic tree topology showed three distinct clusters: YP03-YP04, YP06-YP07-YP05, and YP01-YP02 (Figure 1C,D). This clustering pattern exhibited complete concordance with the hierarchical similarity observed in taxonomic composition analysis, indicating conserved community structure–function relationships within each cluster.

### 3.2. Uncultured Diversity and Novel Lineages in Metagenome-Assembled Genomes

**Figure 2:**
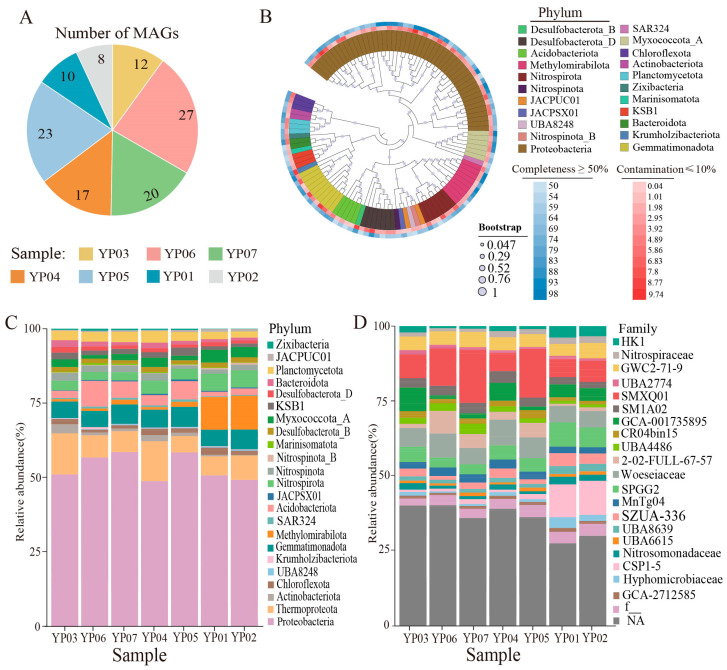
Analysis of microbial community structure and taxonomic composition based on MAGs across seven samples. (**A**) The number of MAGs assembled from each sample. (**B**) Maximum-likelihood phylogenetic tree of the 95 non-redundant MAGs, illustrating their taxonomic affiliation, completeness, and contamination levels. (**C**) Relative abundances of bacterial and archaeal taxonomic composition at phylum level across all samples. (**D**) Relative abundances of bacterial and archaeal taxonomic composition at family level across all samples.

To elucidate microbial functional potential in deep-sea environments, we implemented genome-resolved metagenomics through hybrid assembly and differential coverage binning. This yielded 117 medium-quality metagenome-assembled genomes (MAGs; completeness ≥ 50% and contamination < 10%) with genome sizes spanning 0.76–8.18 Mbp, including 16 high-quality MAGs (completeness ≥ 90% and contamination < 5%) (Figure 2A and Tables S1 and S2). A dereplication pipeline using dRep with 99% average nucleotide identity (ANI) threshold identified 95 non-redundant MAGs representing unique microbial lineages. These MAGs’ classification spans 22 bacterial phyla and Thaumarchaeota according to GTDB-Tk database annotation (Figure 2B and Table S3). Notably, 81.91% of MAGs cannot be classified at the species level, revealing a large amount of unexplored microbial diversity, but this finding still needs further rigorous validation. Proteobacteria dominated the bacterial assemblage (48 MAGs in total), particularly Alphaproteobacteria (21 MAGs) and Gammaproteobacteria (27 MAGs). This distribution pattern is congruent with abyssal plain communities in the eastern Pacific’s Clarion-Clipperton Zone [22]. Subdominant phyla included Desulfobacterota (8 MAGs), Gemmatimonadota (8 MAGs), Nitrospirota (7 MAGs), and Acidobacteriota (5 MAGs). Archaeal MAGs were exclusively affiliated with Thaumarchaeota, representing 5.5–13.88% of community abundance across samples (Figure 2C).

The MAG abundance profiles revealed three distinct groups: YP03-YP04, YP06-YP07-YP05, and YP01-YP02 (Figure 2C,D), mirroring the beta-diversity patterns from PCoA and UPGMA clustering analyses (Figure 1C,D). Seventeen high-abundance MAGs accounting for >50% cumulative abundance were identified (Figure S1), dominated by Thaumarchaeota (Nitrosopumilaceae), Proteobacteria (Gammaproteobacteria), and Desulfobacterota_B. The most abundant MAG (YP07.Bin9; 8.87% relative abundance) was classified as a novel *Nitrosopumilaceae* species within Thaumarchaeota, suggesting key biogeochemical roles in ammonia oxidation.

### 3.3. Metabolic Partitioning of Biogeochemical Cycles Across Deep-Sea Ecosystems

**Figure 3:**
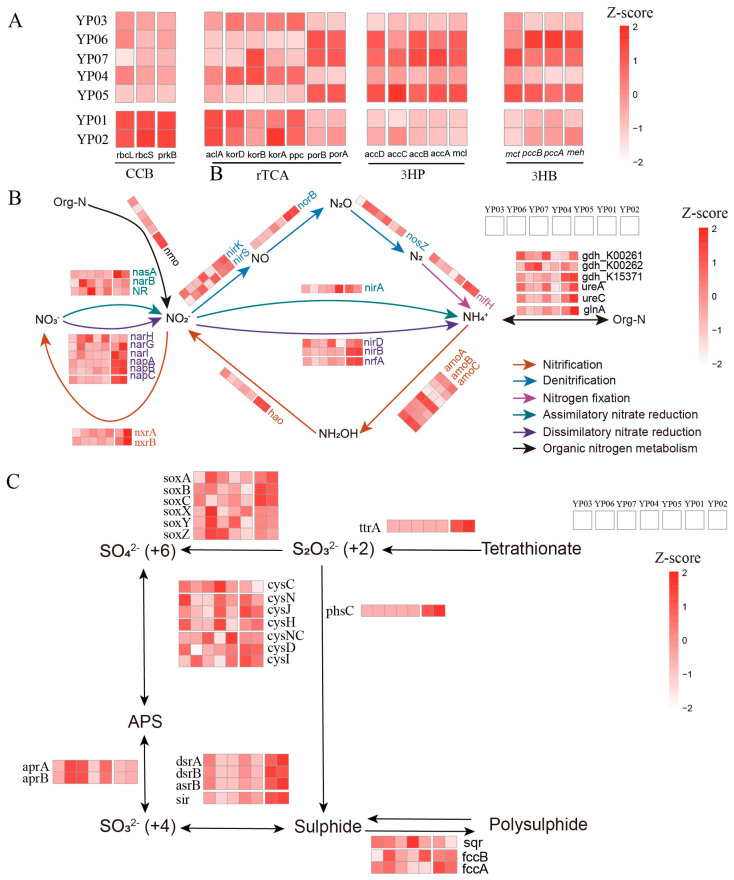
Functional potentials of microbiomes across seven sediment samples. Heatmaps showing the Z-score normalized relative abundances of functional genes involved in (**A**) carbon cycling, (**B**) nitrogen cycling, and (**C**) sulfur cycling.

To characterize microbial contributions to elemental cycling, we conducted systematic annotation of carbon, nitrogen, and sulfur metabolism genes across MAGs. Nazuna seamount samples (YP01/YP02) showed significant enrichment in key autotrophic markers, including RuBisCO subunits (*rbcL*/*S*), phosphoribulokinase (*prkB*), ATP-citrate lyase (*aclA*), and 2-oxoglutarate decarboxylase (*korA*/*D*) compared to abyssal plains (Figure 3A). Notably, the genes (*accA*/*B*/*C*/*D*, *mcl*) related to the 3-hydroxypropionate (3HP) cycle (a microbial CO_2_ fixation pathway) were overrepresented in YP06/07/05, with MAGs affiliated with Proteobacteria (YP03.Bin43, YP06.Bin56, YP04.Bin56, YP05.Bin20) and Nitrospirota (YP07.Bin20) harboring the highest copy numbers of carbon fixation genes (Table S4). Comparative analyses showed that the 3HP pathway genes were present in 76 MAGs, endowing them with significantly higher carbon fixation potential compared to MAGs harboring the Calvin–Benson–Bassham cycle (CBB) (68) or rTCA (10) pathways, underscoring 3HP’s dominance in deep-sea carbon assimilation. Microbes in Nazuna seamount samples exhibited marked upregulation of dissimilatory nitrate reduction genes (*narI*, *napA*/*B*/*C*, *nirB*/*D*, *nrfA*) compared to in those the Magellan seamount region, indicating that the dissimilatory nitrate reduction may play more dominant roles in Marcus-Wake seamount ecosystems (Figure 3B). The genes related to denitrification (*nirS*/*K*, *norB*), nitrification (*nxrA*/*B*, *hao*), and organic-nitrogen-processing genes (*nmo*, *ureAC*, *glnA*, *gdh_k15371*) were enriched in microbes from Nazuna seamount samples. In addition, genes associated with sulfur cycling, including those involved in sulfite reduction (*dsrA*/*B* and *sir*), tetrathionate reduction (*ttrA*), thiosulfate disproportionation (*phsC*), sulfide oxidation (*fccA*/*B*), and sulfur oxidation (*soxA*/*B*/*C*), were significantly more abundant in YP01 and YP02 than in other samples.

**Figure 4:**
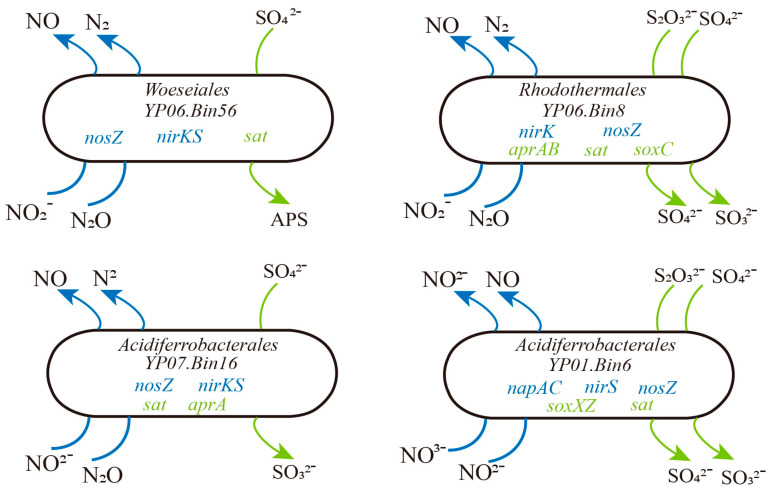
Metabolic characteristics of four S-driven denitrifiers in deep-sea sediments.

Meanwhile, we identified genes related to the coupling of nitrogen and sulfur cycling in the MAGs. Specifically, there are 33 MAGs that all contain genes for both the denitrification and sulfur oxidation pathways, indicating a close coupling between these processes (Table S5). These MAGs with coupled denitrification and sulfur oxidation genes are predominantly found in samples YP06 and YP07. For example, YP06.Bin56, belonging to *Woeseiales*, was a potential novel species. Its genome contained the *nirK*, *nirS*, and *nosZ*, indicating its capacity for complete denitrification, which involves the sequential reduction of nitrate to argon. Additionally, the presence of sat indicated that this bacterium may also have the potential for sulfate reduction. The YP06.bin8 genome contained not only the denitrification-related genes *nirK* and *nosZ* but also the sulfur oxidation gene *soxC* and dissimilatory sulphate reduction genes sat and *aprA*/*B* (Figure 4). This indicated that the bacterium may catalyze the reduction of sulfate to sulfite via sat and further to sulfide via *aprA*/*B*. The presence of *soxC* suggested that it can also oxidize sulfite to sulfate. Hence, we thought these microbes might adapt to deep-sea environments by coupling denitrification with sulfur redox reactions.

### 3.4. Metal Resistance and Cycling Analysis of Microbes Across Deep-Sea Ecosystems

**Figure 5:**
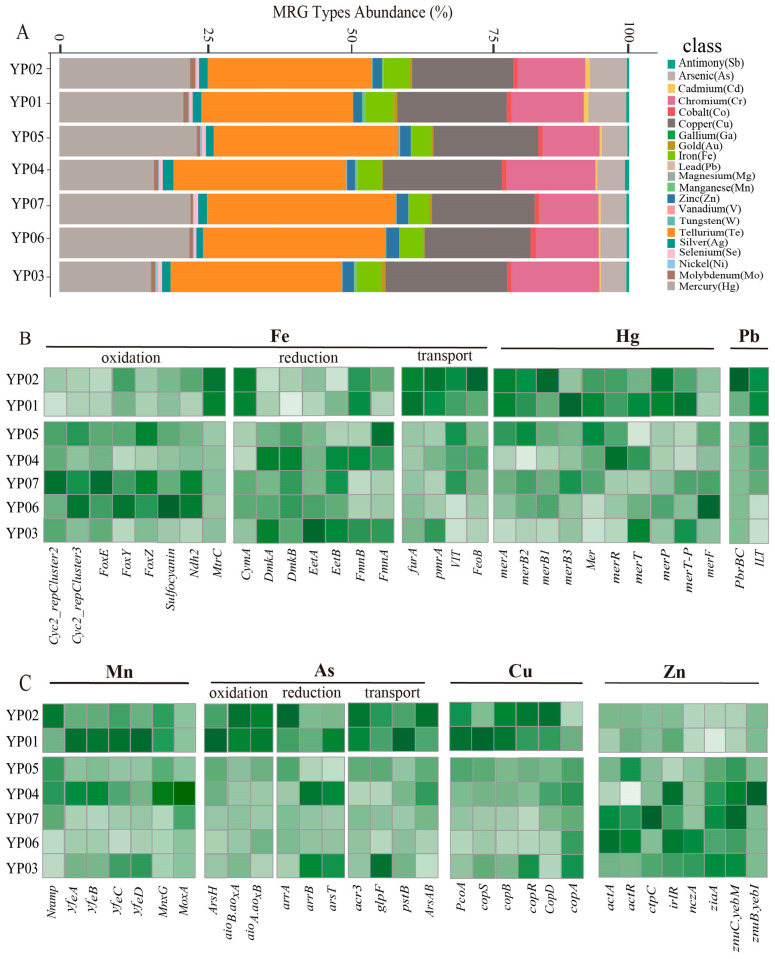
Functional and resistance gene profiles of microbial metal metabolism in deep-sea Sediments. (**A**) Relative abundance of microbial metal resistance genes in seven deep-sea sediment samples based on metagenome reads. The stacked bar chart illustrates the distribution of various metal resistance gene types across the seven samples. Each bar represents a sample, and the segments within each bar denote the relative abundance (%) of specific metal resistance gene types, including copper (Cu), zinc (Zn), cadmium (Cd), and others. (**B**) Heatmaps showing the Z-score normalized relative abundances of functional genes involved in iron (Fe), mercury (Hg), and lead (Pb) metabolism. (**C**) Heatmaps showing the Z-score normalized relative abundances of functional genes involved in manganese (Mn), arsenic (As), copper (Cu), and zinc (Zn) metabolism.

The metal metabolism potential of microbes was thoroughly assessed. A total of 93 metal resistance genes, covering 21 types, were identified across the seven samples based on annotation from the BacMet database. These included genes conferring resistance to manganese (Mn), iron (Fe), copper (Cu), mercury (Hg), zinc (Zn), and other metals (Figure 5A). The most abundant resistance genes were those for tellurium (Te) (ranging from 24.94% to 40.34%) and mercury (Hg), followed by those for copper (Cu), as well as iron (Fe), chromium (Cr), and arsenic (As). Notably, microbial communities in Nazuna seamount samples (YP01 and YP02) displayed significantly higher abundance of iron resistance genes compared to those in the Magellan seamount samples (YP03/04/05/06/07). The aconitase hydratase gene (*acn*) was the most prevalent metal resistance gene (MRG) associated with Fe resistance, followed by *dpsA*, *furA*, and pmrA (Table S6).

The metal metabolic potentials of microbes were evaluated based on marker genes and pathways (Figure 5B,C). The relative abundance of genes involved in iron metabolism varied across the seven samples. Notably, a higher abundance of genes related to iron oxidation was observed in YP06 and YP07, including *Cyc2_repCluster2*, *Cyc2_repCluster3*, *FoxE*/*Y*/*Z*, *Sulfoeyanin*, and *Ndh2*. This suggests active potentials for iron oxidation by microbes in these samples. Genes related to the redox reactions of copper (Cu), arsenic (As), and mercury (Hg), such as Hg (*MerA*/*B*, Hg^2+^ to Hg^+^), copper oxidation (*pcoA*), and arsenic oxidation (*aioA*/*B*/*arsC*), showed higher abundance in the Nazuna seamount samples. Among the Proteobacteria (MAG, n = 6), the highest number of arsenic (As) oxidation genes was found, including *arsH* (n = 5), *aioA* (n = 4), and *aioB* (n = 2), along with arsenic reduction genes, such as *arsR* and *arsC* (MAG, n = 16). Our results indicated that the abundance of arsenic reduction genes in MAGs was significantly higher than that of arsenic oxidation genes (Table S7).

**Figure 6:**
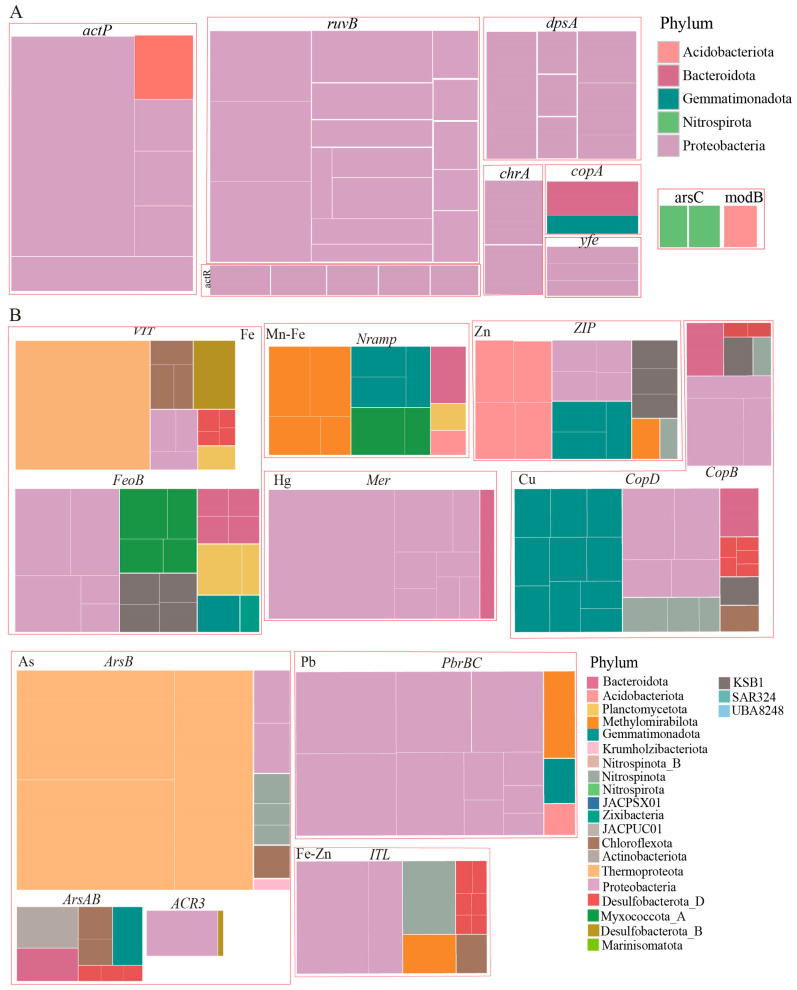
Metal resistance, transport, redox reactions in MAGs. (**A**) Metal resistance of MAGs. (**B**) Metal transport and redox reactions of MAGs.

In addition to redox reactions, most metals are cytotoxic; microorganisms employ metal transporters to regulate intracellular metal ion concentrations. To explore the metal transport potential of MAGs in our research, we summarized the distribution of metal transport genes at the phylum level (Figure 6). Comparative genomic analysis revealed markedly elevated abundances of ferrous iron transport (*FeoB*) and vacuolar iron transporter (*VIT*) genes in the Nazuna seamount region compared to the Magellan seamount region (Figure 5B). A total of 33 MAGs contained iron transport genes *FeoB*, *ITL*, and *VIT*. The MAGs annotated as Proteobacteria and Desulfobacterota are the most abundant phyla. Only MAGs classified as Desulfobacterota contained both *VIT* and *ITL* (Table S8). Additionally, we analyzed the distribution of genes encoding transporters for other metals, such as Cu (*CopB* and *CopD*), Pb (*PbrBC*), Hg (*Mer*), Zn (*ZIP*), and As (*ArsB*, *ArsAB*, and *ACR3*) in MAGs. We found that the Desulfobacterota also harbored genes related to Cu and As transport. Based on the analysis of metal transport and resistance genes in MAGs, we speculated that microbes maintain intracellular metal homeostasis and effectively counteract metal toxicity by regulating the uptake and efflux systems of metal ions.

### 3.5. Metabolic Interdependencies of Sulfur-Metabolizing Bacteria Using Random Matrix Theory Analysis in Nazuna Seamount

The heatmap of gene abundance revealed that *dsrA*, *dsrB*, *dsrC* (involved in dissimilatory sulfur reduction and oxidation) and *phsC* (involved in sulfur reduction) were significantly more abundant in the Nazuna seamount samples compared to others (Figure 3). Further comparative genomic analysis showed that sulfur metabolism in YP01.bin22, YP01.bin30, YP02.bin10, and YP02.bin21 had a high prevalence, with gene counts of 32, 42, 34, and 35, respectively (Table S9). These genes are primarily involved in dissimilatory sulfate reduction and oxidation pathways (Figure S2). To explore the specific metabolite exchange and interactions among sulfur-metabolizing bacteria from YP01 and YP02, we applied Random Matrix Theory (RMT) on the iNAP2.0 platform. This analysis identified potentially transferable metabolites between these MAGs and others, providing insights into potential microbial interactions.

**Figure 7:**
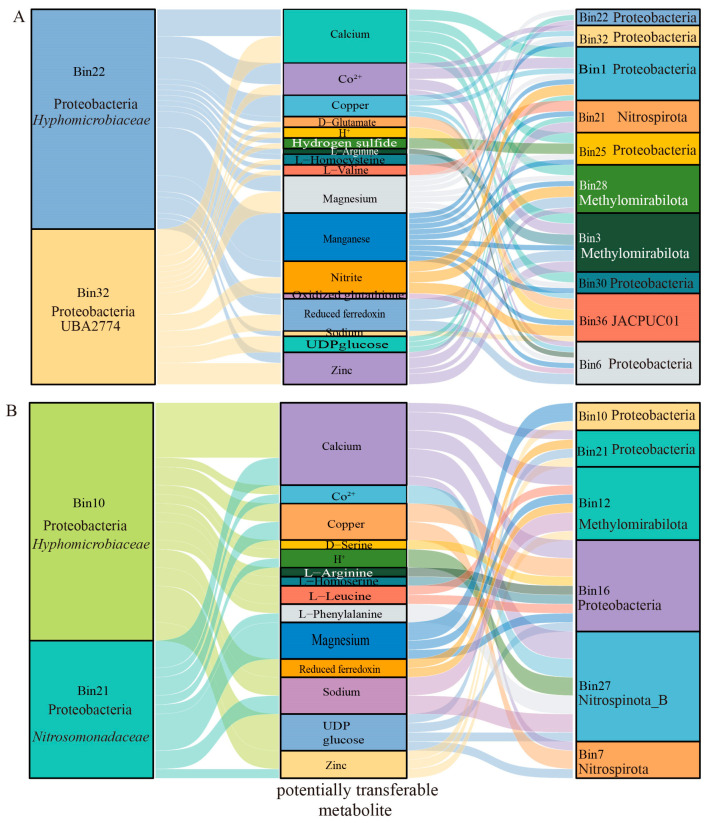
Sankey diagram showing metabolic interactions among sulfur-metabolizing bacteria with other bacteria from seamount samples. Metabolic interactions between sulfur-metabolizing bacteria (YP01.Bin22 and YP01.Bin32) and other MAGs within the YP01 sample (**A**), and those between sulfur-metabolizing bacteria (YP02.Bin10 and YP02.Bin21) and other MAGs within the YP02 sample (**B**). The first and third columns represent the MAGs, while the middle column represents the metabolites involved in the interactions. The width of the lines indicates the relative abundance of the metabolite transfer, with wider lines representing higher transfer rates.

At the family level, YP01.bin22 and YP02.bin10 were annotated as *Hyphomicrobiaceae*, a group of sulfur-oxidizing purple nonsulfur bacteria. The YP02.bin21 and YP01.bin30 belong to Proteobacteria and Desulfobacterota, respectively, the latter being a significant group of sulfate-reducing bacteria. We focused on the potential interactions of these four MAGs in metal metabolism as key exchangeable metabolites that underlie mutualistic dependencies among microbial strains in seamount environments. The results showed that YP01.bin22, YP01.bin30, YP02.bin10, and YP01.bin21 harbored various metal-related potentially transferable metabolites, such as Co^2+^, magnesium, copper, manganese, reduced ferredoxin, and zinc (Figure 7). Among these metabolites, manganese was identified as the most potentially transferable metabolite between YP01.bin22 and other microbial genomes (MAGs), including Bin32, Bin1, Bin21, Bin25, Bin28, Bin3, Bin30, and Bin36. Additionally, reduced ferredoxin linked YP01.bin22 with Bin30, Bin36, and Bin3, indicating potential interactions mediated by reduced ferredoxin. Furthermore, magnesium, reduced ferredoxin, and zinc were recognized as primary potentially transferable metabolites for YP01.bin32 with other MAGs (Figure 7A). Apart from metal metabolites, calcium, H^+^, amino acids, and carbohydrates also played crucial roles as mediators in microbial interactions (Figure 7A,B). Based on these findings, we speculated that microbial communities in seamount environments may integrate sulfur metabolism with metal metabolism to produce metal sulfides, adapting to the high concentrations of heavy metals present in these regions.

## 4. Discussion

Microorganisms in deep-sea sediments remain largely unknown due to the complexity of sedimentary communities and the challenging environmental conditions that hinder their isolation [43]. The unique geochemical environments of different deep-sea geographic structures likely shape distinct microbial communities [44]. In the present study, we identified a new metabolic zonation phenomenon driven by seamount topography. Microorganisms from the Nazuna Seamount in the Marcus-Wake Seamount chain exhibit a stronger autotrophic potential, primarily utilizing the 3-hydroxypropionate (3HP) pathway for carbon fixation, whereas microorganisms from the Magellan Seamount region focus more on nitrification and organic nitrogen metabolism. We also revealed a novel ecological niche construction driven by nitrogen-sulfur coupling metabolism. Thirty-three MAGs were identified to harbor both denitrification and sulfur oxidation genes, suggesting that these microorganisms may adapt to the deep-sea environment by coupling nitrate reduction with sulfur oxidation processes. Furthermore, we uncovered habitat-specific adaptive mechanisms for metal resistance. Microorganisms from the Nazuna Seamount show a significantly higher abundance of iron resistance genes, which aligns with the iron-rich environment characteristic of the seamount. Through random matrix theory analysis, we identified manganese and reduced ferredoxin as the key metabolic exchange molecules in microbial interactions, proposing a novel manganese-mediated microbial interaction mechanism. Finally, MAG analysis revealed a high degree of novelty in seamount microbiomes, with 81.91% without classification annotation of this level, significantly higher than the typical 40–60% novel species observed in deep-sea sediment studies. This suggests that seamounts may serve as biodiversity “hotspots” for microorganisms.

Exploring the diversity of microbial communities in deep-sea sediments is essential for enhancing our understanding of their ecological roles in driving metabolic processes [44,45]. In agreement with that reported in previous studies, the dominant bacterial phyla included Proteobacteria (particularly Alpha-, Gamma-, and Zetaproteobacteria), Chloroflexota, Planctomycetota, and Acidobacteriota, with Proteobacteria being the most abundant (33.18–40.35%). Among archaea, Thaumarchaeota were the most enriched (5.98–10.86%) (Figure 1A). Through assembly and binning, 117 metagenome-assembled genomes (MAGs) were recovered, primarily dominated by Proteobacteria (Alpha- and Gammaproteobacteria), Thaumarchaeota, Gemmatimonadota, Acidobacteriota, Nitrospirota, and Methylomirabilota. These findings align with previous studies based on 16S rRNA or metagenomic sequences from similar habitats [46,47]. The predominance of Proteobacteria as the main prokaryotic group is consistent with records from the Magellan seamount [48]. Gammaproteobacteria and Alphaproteobacteria accounted for nearly half of the total microbial abundance, likely due to their specific adaptations to organic inputs [49]. Notably, Nitrospirota were highly abundant in our samples, particularly in seamount sediments (Figure 2C). There was an unprecedented dominance of Nitrospirota in seamounts (YP01/02; Figure 2C), contrasting with their minor abundance in western Pacific seamount sediments [50] but echoing their niche in ferromanganese crusts [51]. This suggests seamounts are underexplored hotspots for microbially driven Mn cycling. Additionally, significant differences in microbial relative abundance were observed between the Nazuna seamount and the Magellan seamount. For instance, Methylomirabilota, a group of methane-oxidizing bacteria, were significantly more abundant in the Nazuna seamount than in the Magellan seamount (Figure 2C). Methylomirabilota is thought to catalyze the dissimilatory reduction of nitric oxide (NO) to oxygen (O_2_) and nitrogen gas (N_2_) via nitric oxide reductase [52,53]. Overall, these findings indicate distinct microbial community compositions and relative abundances in two seamount regions, with clear spatial distribution patterns and certain similarities across the seven samples. Understanding the functional roles of microorganisms in these habitats is crucial. In this study, metagenomics combined with binning technology was employed to investigate the interactions among key microorganisms and their involvement in carbon, nitrogen, sulfur, and metal metabolic pathways across different deep-sea geographic structures. Previous research has demonstrated that chemoautotrophs utilize the Calvin–Benson–Bassham cycle (CBB) and the Wood–Ljungdahl pathway (WL) to assimilate carbon dioxide (CO_2_) [54]. In contrast to the Calvin cycle found mainly in the hydrothermal area [55], our MAGs revealed that microbiomes in the Nazuna seamount exhibited heightened autotrophic potential via the 3-hydroxypropionate pathway. Our analysis revealed that MAGs classified as Methylomirabilota in the Nazuna seamount samples harbored a higher number of genes, such as *rbcL*, *rbcS*, and *prkB*, associated with carbon fixation (Figure 3A and Table S3). Regarding the nitrogen cycling of microbes, genes linked to dissimilatory nitrate reduction, denitrification, nitrification, and organic nitrogen metabolism were more abundant in the Nazuna seamount compared to the Magellan seamount (Figure 3B). Chemoautotrophic microorganisms generate energy by completely oxidizing thiosulfate or sulfide to sulfate, a critical mechanism for energy acquisition in anaerobic environments [12]. In Nazuna seamount microbes, genes involved in sulfite reduction (*dsrA*, *dsrB*) and sulfur oxidation (*soxA*, *soxB*, *soxC*) were more abundant than in other samples (Figure 3C). Multifunctional microorganisms, including methanogens, ammonia-oxidizing archaea (AOA), and sulfate-reducing bacteria (SRB), have been confirmed to possess nitrogen-fixing capabilities [56]. Nitrate-reducing sulfide oxidizers can couple sulfide oxidation with nitrate reduction [57]. The coupling of denitrification and sulfur oxidation may contribute to microorganisms in detoxifying sulfides in sediments [58]. In our study, YP06.Bin56, a potential novel species of *Woeseiales*, contains genes for complete denitrification and potential sulfate reduction. YP06.bin8 possesses denitrification-related genes alongside sulfur oxidation and dissimilatory sulfate reduction genes, indicating its ability to catalyze sulfate reduction to sulfide and sulfite oxidation to sulfate (Figure 4). Collectively, our findings underscore the ecological significance of sulfur oxidation and sulfate reduction in these environments. Although our study provides genomic evidence for the coupling of denitrification and sulfur oxidation, future research will focus on transcriptomic and proteomic analyses to validate these findings at the functional level. These methods will help confirm the expression of key genes involved in these processes and provide a more comprehensive understanding of their ecological roles. Experimental validation of these metabolic pathways will also be crucial for confirming the adaptive mechanisms of these microorganisms in extreme environments.

To explore the potential of microbial metal metabolism, analysis based on the MRGs database revealed that microorganisms in the 7 samples harbored 21 types of metal resistance genes (e.g., Fe, Cu, Hg, As, etc.), with tellurium (Te) and mercury (Hg) resistance genes being the most abundant, followed by copper (Cu), iron (Fe), chromium (Cr), and arsenic (As) resistance genes. The abundance of iron resistance genes was significantly higher in seamount samples (YP01 and YP02) compared to plain samples (Figure 5A), suggesting adaptation to iron-rich inputs characteristic of seamount environments, where microbial iron oxidation may contribute to mineral precipitation and biofilm formation. Studies have shown that microorganisms can transform highly toxic metals into less toxic forms through oxidation and reduction reactions [59,60]. For instance, *Sulfurimonas marisnigri* (isolated from the Black Sea) mediates sulfide oxidation coupled with Mn (IV) reduction, completely oxidizing sulfide to sulfate [61]. In our results, genes involved in metal redox reactions, (e.g., *Cyc2_repCluster2*, *Cyc2_repCluster3*, *FoxE*/*Y*/*Z*, *Sulfoeyanin*, and *Ndh2*) were more abundant in samples YP06 and YP07, potentially supporting chemolithoautotrophic growth in sulfur-rich niches. Conversely, mercury reduction genes (*merA*/*B*), copper oxidation genes (*pcoA*), and arsenic oxidation genes (*aioA*/*aioB*/*arsC*) were enriched in YP01 and YP02, indicating niche-specific strategies for detoxifying volcanically derived metals in seamounts.

The phylum Proteobacteria carried the highest number of arsenic oxidation genes (*arsH*, *aioA*, and *aioB*), as well as arsenic reduction genes (*arsR* and *arsC*) (Figure 5B,C), likely enabling dominance in arsenic-contaminated zones through energy conservation (e.g., arsenite oxidation) and efflux-based detoxification. To maintain metal ion homeostasis, microorganisms utilize metal transport proteins to shuttle metals across cellular compartments [62]. Efflux mechanisms are critical for bacterial resistance to multiple heavy metals [63]. Under anaerobic or microaerobic conditions, FeoB, situated in the periplasmic membrane, mediates the uptake of free ferrous iron (Fe^2+^) and transfers these iron ions to the cytoplasm in a GTP-dependent manner [64]. The abundance of iron ion transport genes *FeoB* and *VIT* was significantly higher in seamount samples than in plains. Among the MAGs, twelve contained *VIT* genes, which were phylogenetically assigned to Proteobacteria (three MAGs), Desulfobacterota (five MAGs), and Chloroflexota (two MAGs). The prokaryotic membrane protein FeoB, essential for Fe (II) uptake in bacteria [65], was identified in 14 MAGs and found across Proteobacteria, Bacteroidota, KSB1, and Myxococcota_A (Figure 6B). We speculated FeoB and VIT facilitate Fe^2+^ influx for cellular demands, while efflux systems (e.g., czcA, copA) prevent cytoplasmic overload. These systems collectively enable survival in dynamic redox gradients.

Furthermore, microorganisms are interconnected through the exchange of compounds, energy, and information, forming complex interactions such as competition, mutualism, and commensalism [66,67]. Sulfur compounds serve as both electron donors and acceptors, enabling sulfur-oxidizing microorganisms (SOBs) and sulfur-reducing microorganisms (SRBs) to facilitate electron transfer via redox reactions. This process, coupled with metal metabolism, leads to the formation of inert and insoluble sulfides, enhancing microbial adaptation and contributing to the remediation of heavy metal pollution. For example, the strain *Halomonas* sp. MNB13, isolated from deep-sea nodules, utilizes cysteine metabolism to generate H_2_S, scavenge reactive oxygen species (ROS), and mitigate Mn^2+^ toxicity [16]. In our study, the sulfur-metabolizing microorganisms YP01.bin22, YP01.bin30, YP02.bin10, and YP01.bin21 carried various metal-related transferable metabolites, including Co^2+^, magnesium, copper, manganese, reduced ferredoxin, and zinc (Figure 7). Among these, manganese was identified as the most transferable metabolite between YP01.bin22 and other microbial genomes (MAGs). In addition to metal metabolites, calcium, H^+^, amino acids, and carbohydrates also played crucial roles as mediators in microbial interactions (Figure 7A,B). Microbial interaction networks further reveal Mn^2+^ as the dominant transferred metal metabolite—distinct from Fe-centric interactions in the tangyin hydrothermal vents [68]. This underscores seamounts as hubs for manganese-mediated syntrophy, a previously overlooked ecological driver. This study highlights the diversity and functional complexity of deep-sea microbial communities, emphasizing their critical ecological roles in deep-sea environments. These findings provide a scientific basis for the conservation and sustainable utilization of deep-sea ecosystems.

## 5. Conclusions

This study provides valuable insights into microbe composition, diversity, and metabolic potential in the Marcus-Wake and Magellan Seamount regions. Bacteria, predominantly Proteobacteria, Chloroflexota, and Planctomycetota, dominated over archaea (mainly Thaumarchaeota). Metagenome-assembled genomes (MAGs) revealed 81.91% novel species, indicating a large amount of unexplored microbial diversity. Distinct microbial abundance and clusters highlight the microbial community groups by seamount region, indicating localized biodiversity with high endemism. Microbes in the Nazuna seamount exhibited significant potential for carbon fixation via the 3HP pathway, dissimilatory nitrate reduction, and sulfur cycling, suggesting key roles in biogeochemical processes. The identification of nitrogen-sulfur coupling and metal resistance genes, especially in the Nazuna seamount microbes, indicated adaptive strategies to high metal concentrations. Microbe harbor widespread metal resistance genes, with notable differences in iron resistance gene abundance between the Nazuna and Magellan seamount samples. Microbes likely maintain metal homeostasis through regulated ion transport systems. In the Nazuna seamount, Random Matrix Theory analysis revealed complex interactions among sulfur-metabolizing bacteria, involving metal and sulfur metabolism. These interactions may enable microbes to adapt to high heavy metal concentrations by producing metal sulfides. Overall, this study enhances our understanding of microbial community structures, metabolic functions, and adaptation strategies in deep-sea seamount ecosystems, underscoring their importance in biogeochemical cycling and ecosystem function.

## Data Availability

The metagenome sequencing data were submitted to the Genome Sequence Archive at the National Genomics Data Center, China National Center for Bioinformation/Beijing Institute of Genomics, Chinese Academy of Sciences. The metagenome sequencing data is CRA024169 (https://ngdc.cncb.ac.cn/gsa/browse/CRA024169, accessed on 28 March 2025).

## Supplementary Materials

The following supporting information can be downloaded at: https://www.mdpi.com/article/10.3390/microorganisms13071467/s1. Figure S1: MAGs are ordered by their average relative abundance across all samples. Dominant MAGs, whose cumulative relative abundance accounts for at least 50% of the total, are indicated by dots on the x-axis. Figure S2: Number of genes involved in sulfur oxidation and reduction in each MAG. Table S1: Sample point information and metagenome information. Table S2: Genomic information of bins. Table S3: Species annotation information of bins. Table S4: Carbon metabolic in MAGs. Table S5: Nitrogen and sulfur metabolic in MAGs. Table S6: Metal resistance gene in MAGs. Table S7: Arsenic metabolism of gene in MAGs. Table S8: Metal metabolism of gene in MAGs. Table S9: Carbon, nitrogen, and sulfur metabolism of gene in MAGs.
