# Supplementary figures and images for: Comparative Metagenomics Reveals Microbial Diversity and Biogeochemical Drivers in Deep-Sea Sediments of the Marcus-Wake and Magellan Seamounts
Source: Microorganisms. 2025 Jun 24;13(7):1467. doi: 10.3390/microorganisms13071467 (PMC12300337; doi:10.3390/microorganisms13071467)

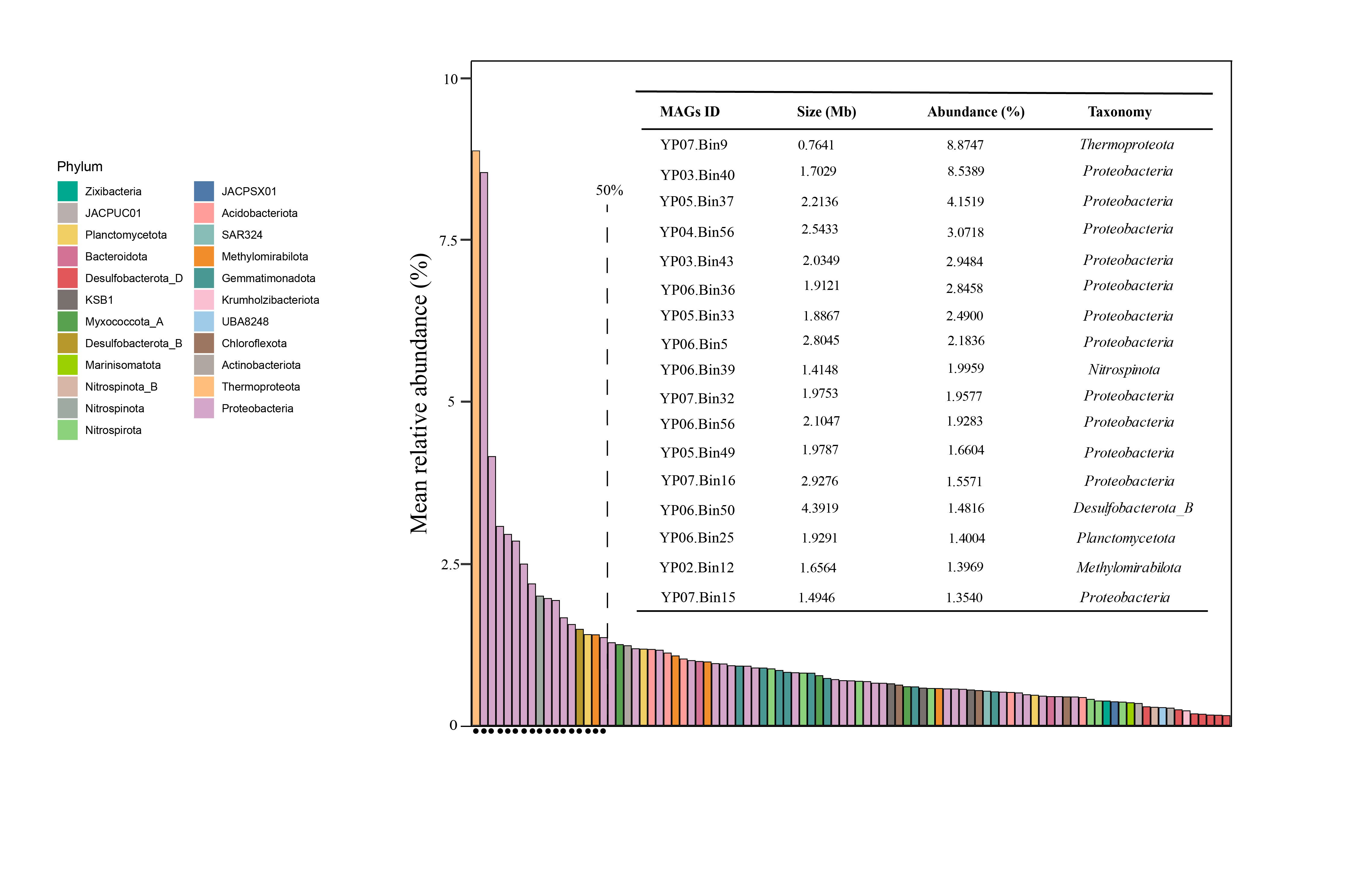

Supplement: Supplementary file 1 [file microorganisms-13-01467-s001.zip › Figure S1.jpg]

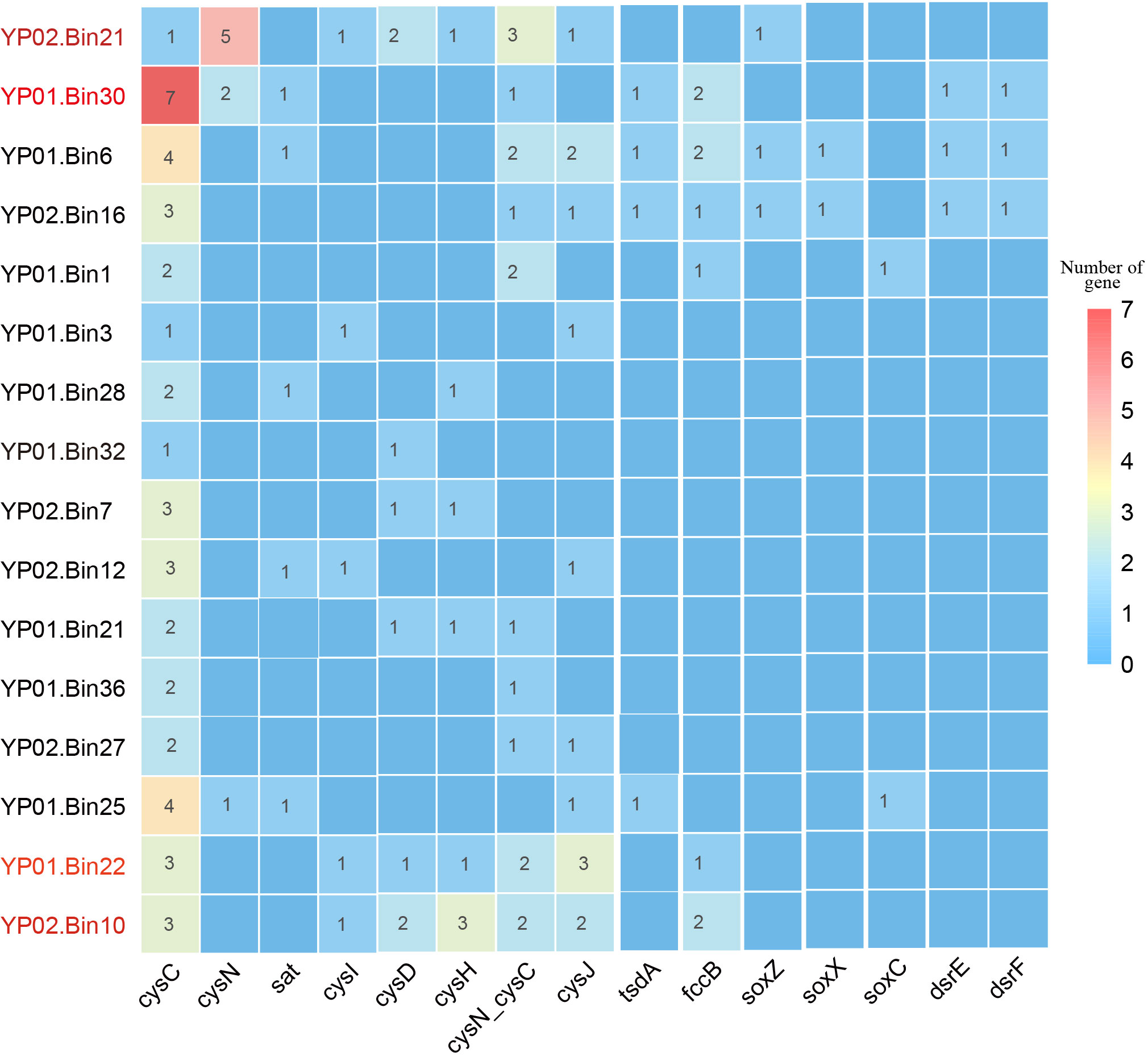

Supplement: Supplementary file 1 [file microorganisms-13-01467-s001.zip › Figure S2.jpg]
